# Earthquakes and very deep groundwater perturbation mutually induced

**DOI:** 10.1038/s41598-021-92937-y

**Published:** 2021-07-01

**Authors:** Dugin Kaown, Kang-Kun Lee, Jaeyeon Kim, Jeong-Ung Woo, Sanghoon Lee, In-Woo Park, Daeha Lee, Jin-Yong Lee, Heejung Kim, Shemin Ge, In-Wook Yeo

**Affiliations:** 1 School of Earth and Environmental Sciences, Seoul National University, Seoul, 08826 Korea; 2 Department of Geophysics, Stanford University, Stanford, CA 94305 USA; 3 Department of Geology, Kangwon National University, Chuncheon, 24341 Korea; 4 Geological Sciences, University of Colorado, Boulder, 80309 USA; 5 Department of Geological and Environmental Sciences, Chonnam National University, Gwangju, 61186 Korea

**Keywords:** Environmental sciences, Hydrology

## Abstract

We report unique observations from drilling and hydraulic stimulation at a depth of approximately 4.3 km in two Enhanced Geothermal System (EGS) wells at the Pohang EGS site, South Korea. We surveyed drilling logs and hydraulic stimulation data, simulated pore pressure diffusion around the fault delineated by seismic and drilling log analyses, conducted acoustic image logging through the EGS wells, observed significant water level drops (740 m) in one of the two EGS wells, and obtained hydrochemical and isotopic variation data in conjunction with the microbial community characteristics of the two EGS wells. We discuss the hydraulic and hydrochemical responses of formation pore water to a few key seismic events near the hypocenter. We focused on how the geochemistry of water that flowed back from the geothermal wells changed in association with key seismic events. These were (1) a swarm of small earthquakes that occurred when a significant circulation mud loss occurred during well drilling, (2) the M_W_ 3.2 earthquake during hydraulic stimulation, and (3) the M_W_ 5.5 main shock two months after the end of hydraulic stimulation. This study highlights the value of real-time monitoring and water chemistry analysis, in addition to seismic monitoring during EGS operation.

## Introduction

Induced earthquakes at Enhanced Geothermal System (EGS) sites have been observed globally, but the magnitude of the M_W_ 5.5 event that occurred owing to the rupture of the critically stressed fault at the Pohang EGS site in South Korea was unusual; this event corresponded to a “run-away” earthquake^1–3^. The terms “run-away” or “triggered” earthquake have been used to describe rupturing beyond the volume affected by stimulation^4^. Water injected under pressure into deep aquifer systems has induced a number of earthquakes in many places^5–11^. For instance, low pressure injection in geothermal fields induced a M_W_ 4.6 earthquake at the Geysers field in northern California in the 1980s^7^. Furthermore, the injection of water at a high pressure into a tight hot dry rock at an EGS site in Basel, Switzerland induced series of earthquakes in 2006 and 2007, with the largest event being a M_L_ 3.4 event^12^. Other examples include brine water injection into a 4.3-km-deep well in Paradox Valley, Colorado, USA, which induced more than 1,500 M ≥ 1 earthquakes, including a M_W_ 3.9 earthquake in 2013^5,13^, and wastewater injection in Oklahoma, USA, which has increased the seismicity of the region since 2008 and induced M_W_ 5.7 and 5.8 earthquakes in 2011 and 2016^6,14,15^, respectively.

**Figure 1:**
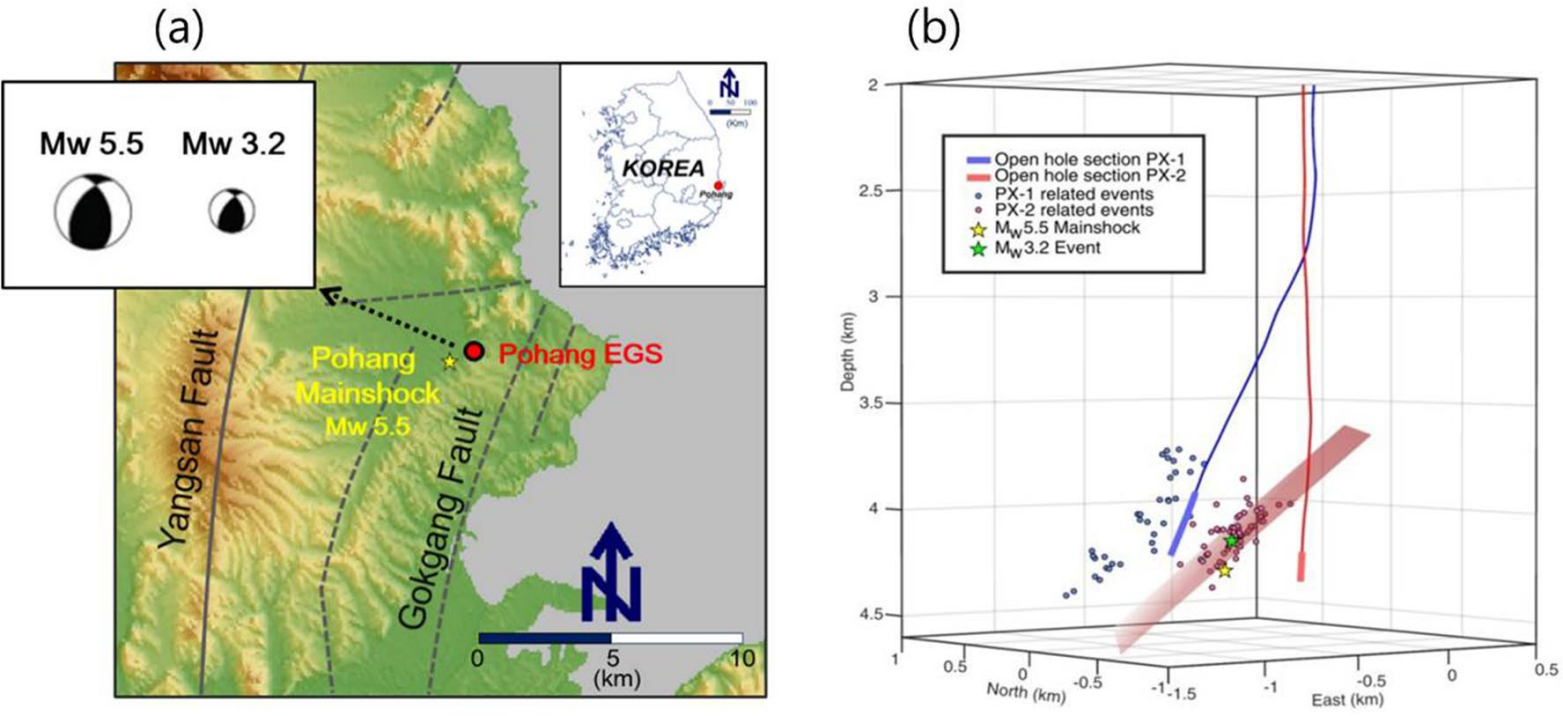
(**a**) Location map of the Pohang Enhanced Geothermal System (EGS) site, South Korea. Map was generated using Generic Mapping Tools (GMT) software, version 4.5.9^47^ (http://gmt.soest.hawaii.edu/projects/gmt). (**b**) a perspective view of the earthquakes related with the events at PX-1 and PX-2.

The November 15, 2017 Pohang earthquake was South Korea’s second strongest earthquake in magnitude (M_W_ 5.5) and the most destructive earthquake since instrumental observation began in 1978^16^. Adjacent to the epicenter, a geothermal project to develop an EGS was operational. The experimental EGS site is located in the Pohang area, which exhibits a relatively higher heat flow and geothermal gradient compared with other areas in South Korea ^16–19^ (Fig. 1a). During EGS development, two geothermal wells were drilled to a depth of approximately 4.3 km, and hydraulic stimulation tests were conducted by injecting water under high pressure into the wells^2^. The focal depths of the M_W_3.2 and M_W_5.5 earthquakes were estimated as 4.146 and 4.270 km, which were in close proximity to the damaged casing of 3.8 km^20^. After the main shock, an abrupt water level deviation between wells PX-1 and PX-2 of approximately 740 m occurred. Hydrological responses after earthquake events have also been reported in several studies^21–29^. For example, an increase in the streamflow was observed after the 2003 San Simeon (California) Earthquake^27^ while other studies identified new streams and springs that originated from groundwater in adjacent mountains after the 2014 South Napa M_W_ 6.0 earthquake in California, USA^30^. Increased stream discharge was also observed after a M_W_ 5.8 injection-induced earthquake near Pawnee, Oklahoma, USA, in 2016^24^. Kaown et al.^21^ and Kim et al.^22,31^ combined hydrologic, hydrochemical, and isotopic analyses to explain such earthquake-induced water levels and hydrochemical changes in the groundwater at a depth range of 100 m. During hydraulic stimulation at the Pohang EGS site, Westaway et al.^32^ compared the chemical constituents of the injected water and flowback water, indicating the possible degradation of the fault strength through hydrochemical reactions. These observations, however, were mostly obtained from surface or subsurface environments shallower than several hundred meters in depth or for a short time before the earthquake. To provide new insights into changes at greater depths before and after the earthquake, this study provides observations from a depth of 4.3 km, where one of the EGS wells was intersected by a fault, which ruptured and led to the M_W_ 3.2 and 5.5 seismic events in April and November 2017, respectively. The objectives of this study were (1) to report unique observations of (i) high-pressure water injection and flowback through two deep EGS wells, (ii) two injection-induced earthquakes and huge water level drop at an EGS well, and (iii) subsequent earthquake-initiated deep pore water perturbations near the EGS wells; and (2) to explore co-seismic and post-seismic interactions between deep pore water and earthquakes using hydraulic, chemical, and microbial signatures.

## Results

### Key seismic events and deep pore water perturbations

**Figure 2:**
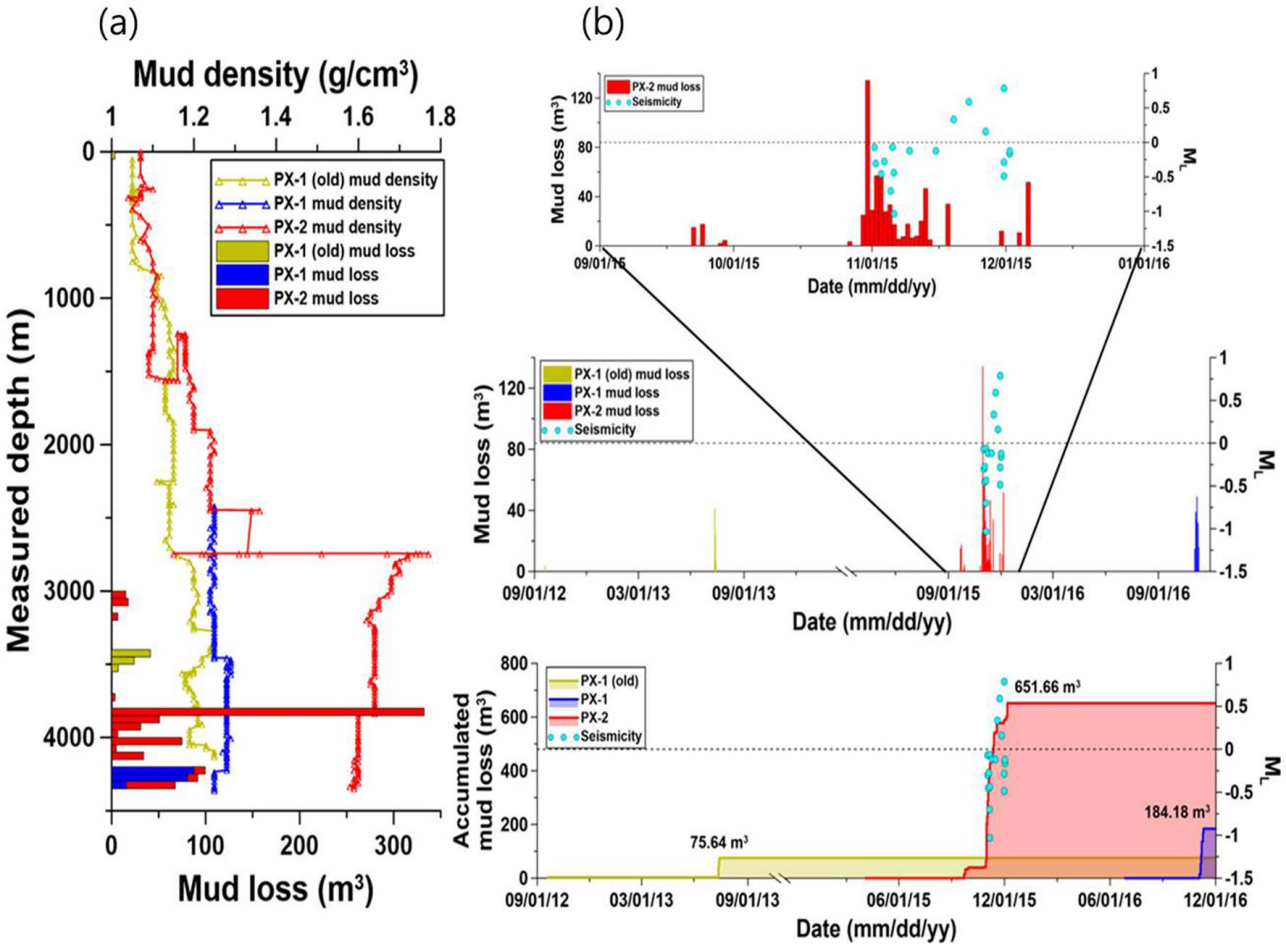
(**a**) Volume of mud loss and mud density of the PX-1 (old), PX-1, and PX-2 wells at specific depths during the drilling periods and (**b**) the temporal distribution of accumulated mud loss and seismicity.

Until two months prior to the M_W_ 5.5 mainshock on November 15, 2017, the fault that eventually ruptured during the mainshock had been continuously pre-stimulated by five hydraulic stimulations and mud circulation, which led to mud loss while drilling the two EGS wells (Fig. S1). The first series of seismicity appeared when significant loss circulation (mud loss) occurred at the end of October 2015 (Fig. 2). Hydraulic stimulation commenced in January 2016, and five stimulations (each corresponding to a period of a few weeks) continued until September 18, 2017. Prior to the main shock, the largest earthquake (M_W_ 3.2) occurred on April 15, 2017, and resulted in the discharge of not only injected water (surface reservoir water) but also deep formation water through the two EGS wells. Deep formation water discharge, lasting approximately 24 h, indicated that the fault rupture had created a link between the formation water and open-hole section of the EGS well PX-1 at a depth of 4 km.

**Figure 3:**
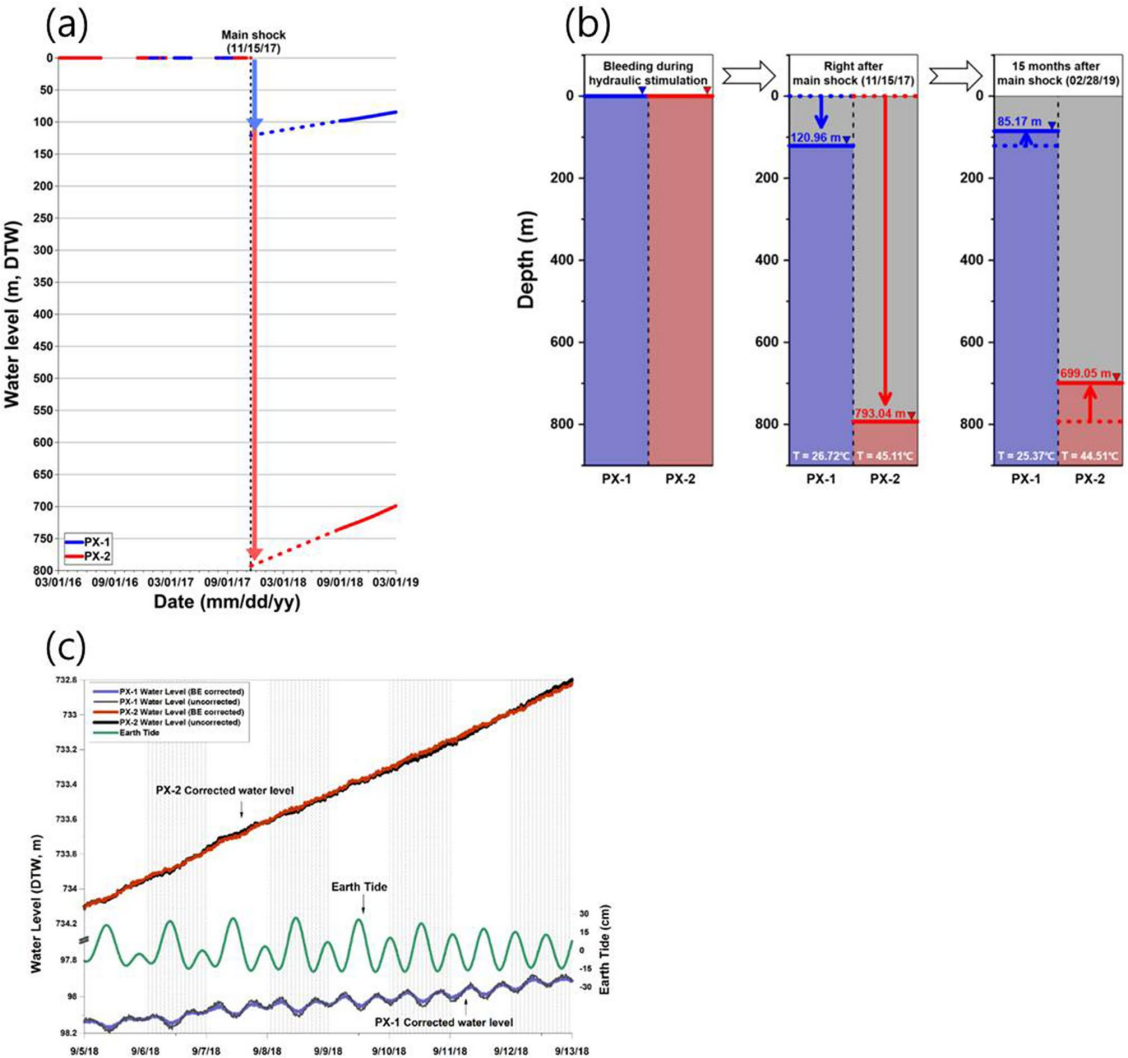
(**a**) Water level deviations between two injection wells after the M_W_ 5.5 event; (**b**) groundwater level data form August 2018 to February 2019 in PX-1 and PX-2, showing that the groundwater level increased at rates of 0.0761 and 0.2000 m/day in PX-1 and PX-2, respectively; and (**c**) time series of the Earth tide vertical component and responses of each well to the Earth tide.

The earthquakes in the period of the hydraulic stimulation into the PX-2 well progressively deepen in the southwestern direction along the fault plane^2,20^. Their hypocenters were firmly resolved by the Double Difference method and the absolute location of a key event that was observed by borehole sensors at the PX-2 well^20^. The absolute error of the key event and average relative location errors were estimated as ≲100 m and < 30 m, respectively. Figure 1b shows that the projected fault plane that best fit the hypocenters of the induced earthquakes corresponded with fault plane solutions of M_W_ 3.2 and 5.5 earthquakes^3,20^. The projected fault plane was further confirmed by additional supporting evidence obtained from acoustic image logging along the second EGS well PX-2^2^. The results indicated that the well casing of PX-2 was damaged or most likely broken by the fault activated during the main shock at an approximate depth of 3.8 km. At this same depth, significant circulation mud loss of more than 650 m^3^ occurred during drilling. This depth corresponds to the point where the projected fault crossed the well. During the M_W_ 3.2 earthquake, water of a completely different quality (when compared with previous discharge) was temporarily discharged from PX-1 immediately after the M_W_ 3.2 earthquake with an extremely short time delay for wellbore-storage water outflow. During the M_W_ 5.5 main shock, a distinct water level drop of more than 740 m occurred in PX-2 (Fig. 3).

### Loss circulation and related seismic events

Based on daily drilling reports, mud loss occurred at specific depths in each EGS well during drilling (Table S1, Fig. 2). However, loss circulation is not typical, which therefore indicates the existence of a highly permeable formation or a fracture zone. The depth of mud loss occurrence was approximately 3.8 km in PX-2 and 4.2 km in PX-1, thus indicating the potential existence of a fault.

Figure 2 shows the mud loss amounts and density of the drilling mud versus depth for PX-1 and PX-2 during the drilling process. Figure 2a indicates that the amount of mud loss for both EGS wells during drilling was concentrated at a depth interval of 3785–3840 m. The densities of the drilling mud were approximately 1.2 g/cm^3^ for PX-1 and 1.6 g/cm^3^ for PX-2. The logging data during drilling suggest that a fault zone existed at the depth where the mud loss volume in PX-2 was high^2^. This mud loss may have resulted in additional pressure exceeding 20 MPa onto the formation due to the 3.8-km column of denser mud in PX-2. Induced earthquakes related to the mud loss are shown in Fig. 2b. Data from the PHA2 seismological station, the nearest permanent station to the EGS site, were used in this study. Seismic events induced by mud loss are unusual and are shown in Table S1. Since the survey period from 2009, microseismicity was not recorded at the EGS site prior to November 2015 (Fig. 2b). However, microseismicity was recorded following the massive loss circulation that occurred in PX-2 on November 1, 2015. Specifically, the distribution of microseismicity was concentrated from November 1 to December 1, 2015. Following this microseismicity, several episodes of earthquakes occurred associated with the hydraulic stimulation experiments (Fig. S1).

### Hydraulic stimulation and M_W_ 3.2 earthquake

The M_W_ 3.2 earthquake halted the second hydraulic stimulation in PX-2 on April 15, 2017 (Fig. S1). One and a half days after the M_W_ 3.2 earthquake, formation water was discharged from both PX-1 and PX-2. The flowback water from PX-1 exhibited a brown color with milky bubbles for more than several hours, while clear water was observed in the backflow water from PX-2 (Fig. S2a). Although the amounts of backflow discharge from PX-1 and PX-2 were nearly identical, the temperatures differed, corresponding to 31.7 °C and 30.3 °C in PX-1 and PX-2, respectively. Both wells had previously exhibited nearly identical temperatures (26.8 °C and 27.4 °C in PX-1 and PX-2, respectively). The increase in the water temperature of PX-1 (4.9 °C) when compared to that of PX-2 (2.9 °C) indicated the mixing of a portion of deeper and warmer formation water into the PX-1 open-hole section at the point where it intercepts the fault that ruptured it during the M_W_ 3.2 earthquake; thus, formation water and dissolved or trapped gases under high pressure were released. The time at which formation water discharge from PX-1 after the M_W_ 3.2 earthquake commenced approximately corresponded to one and half days. This was identical to the time necessary for the wellbore storage (~ 80 m^3^) in PX-1 to discharge. This implies that formation water was introduced into the open-hole section in PX-1 (at an approximate depth of 4049–4362 m) immediately after the M_W_ 3.2 earthquake; furthermore, the time required to discharge this formation water after wellbore storage in PX-1 corresponded to one and half days.

**Figure 4:**
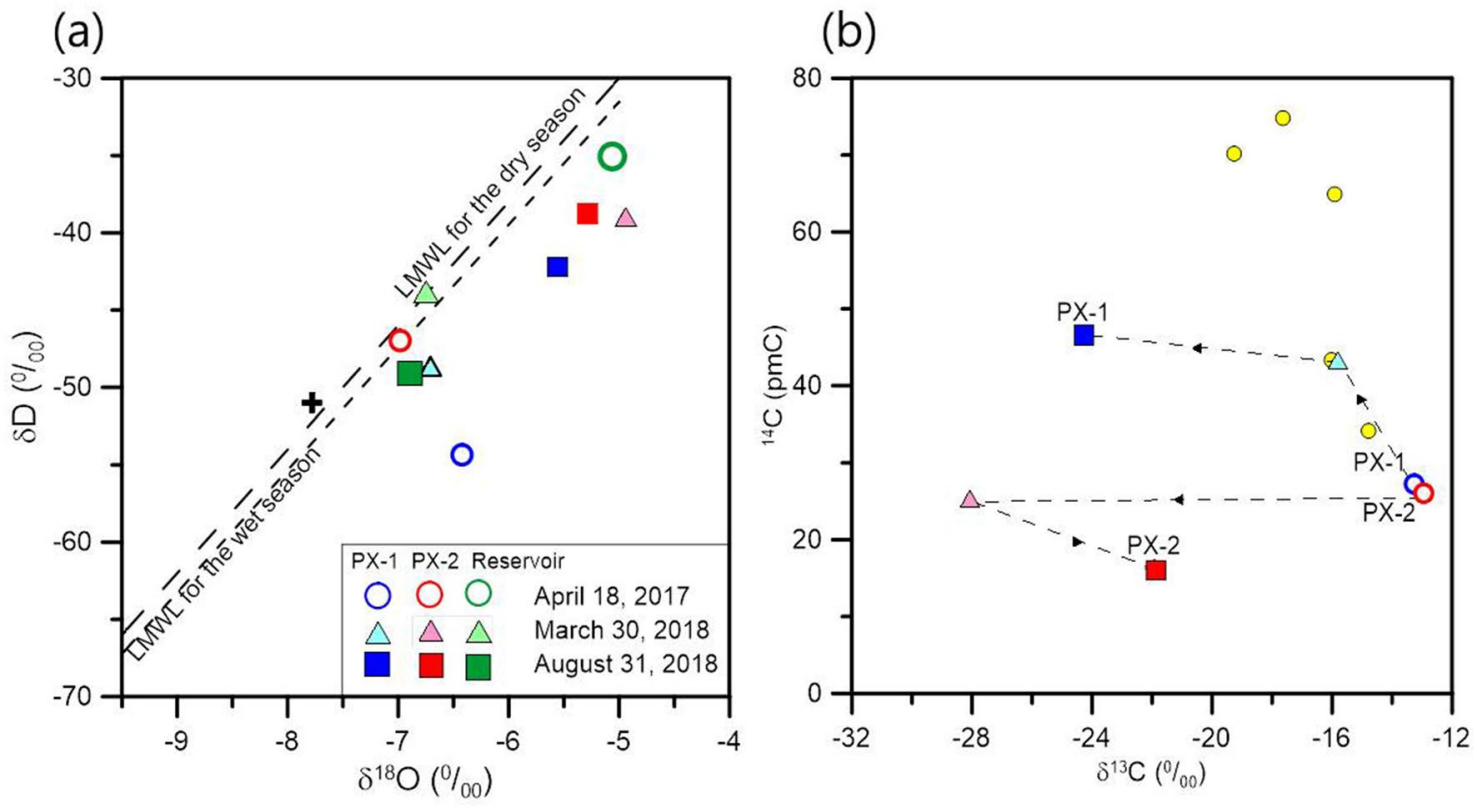
Comparison of the water chemistry of PX-1, PX-2, and reservoir water used as injection water after the M_W_ 3.2 and 5.5 earthquakes in (**a**) δ^18^O and δD; and (**b**) ^14^C and δ^13^C; yellow circles indicate samples collected near the Pohang area described in Kaown et al.^21^. The black cross indicates a weighted average of δ^18^O and δD in precipitation data around the Pohang area by Lee and Chung^48^.

The chemical and isotopic properties of backflow from PX-1 and PX-2 were also different from each other (Fig. 4a), as follows. The values of δ^18^O and δD in PX-2 exhibited less deviation from the local meteoric water line (LMWL) compared to those of PX-1 because hydraulic stimulation was conducted in PX-2 using the water from the surface reservoir water (Fig. 4a). The values corresponding to δ^18^O and δD in PX-1 deviated from the LMWL, thereby suggesting the characteristics of deep formation water. Formation water exhibited increased values of δ^18^O due to the increase in water–rock interactions under high temperatures and pressures at relevant depths^17,33–35^. The chemical characteristics of the backflow from PX-1 and PX-2 were compared on a Piper diagram (Fig. S2b). Backflow from PX-2 exhibited a composition similar to that of the surface reservoir water.

### M_W_ 5.5 earthquake and extreme water level drop

**Figure 5:**
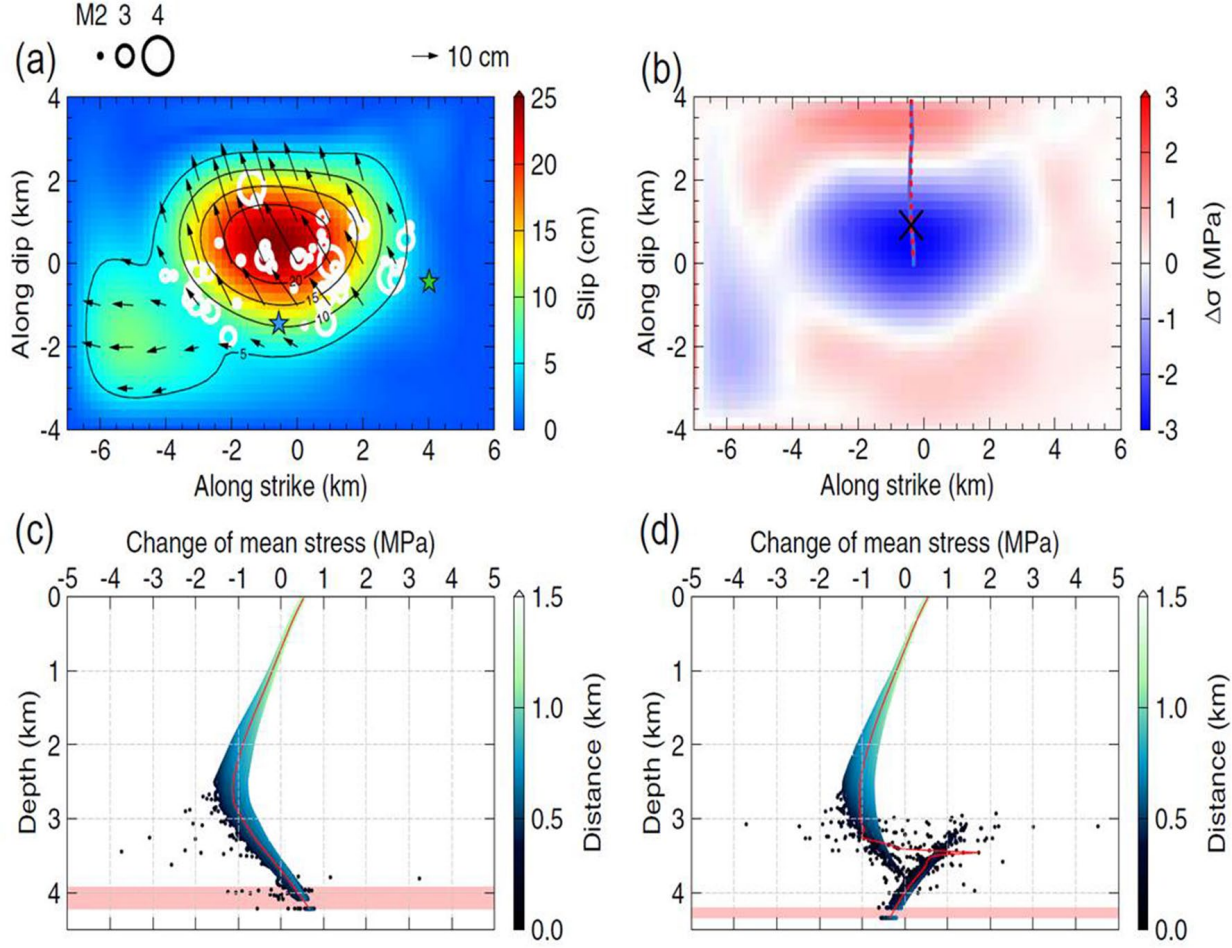
(**a**) Spatial distribution of cumulative slip for the Pohang mainshock. Black arrow indicates the cumulative slip vector, of which the amplitude is greater than 5 cm. Aftershocks whose magnitudes are greater than 2 are projected on the fault plane as white circles and M_L_ 4.3 and 4.6 aftershocks are denoted as blue and green stars. (**b**) Stress drop estimated from the finite rupture model of (**a**). Projections of the crossing point of PX-2 and the trajectories of PX-1 and PX-2 are illustrated as an x symbol, solid blue lines, and red-dashed lines, respectively. (**c**, **d**) Changes in the mean stress with depth for PX-1 (**c**) and PX-2 (**d**). The changes in the mean stress at their trajectories are illustrated as red lines and those for the horizontally shifted trajectories are colored with the offset from the assumed fault plane. The open hole intervals are colored in pale red. In (**a**) and (**b**), the origin of the coordinate is set to the location of the main shock.

The M_W_ 5.5 main shock occurred two months after the fifth and final hydraulic stimulation in PX-2 on November 15, 2017. Well PX-1 was maintained in the shut-in phase until the earthquake after the final stimulation. After the M_W_ 5.5 earthquake, well PX-1 was opened and water flowed back to the surface through the well immediately after the M_W_ 5.5 earthquake until January 4, 2018. However, no water flow back was observed in PX-2. Acoustic image logs, in conjunction with water level measurements, were conducted nine months after the M_W_ 5.5 earthquake in August 2018. Groundwater levels decreased to 740 m below the surface in PX-2 while the water level in PX-1 was 113 m below the surface (Fig. 3). The significant water level drop in PX-2 indicated that at least 20 m^3^ of water in the wellbore was discharged into the formation, possibly through the ruptured fault because the ruptured plane passed through PX-2 at a depth of 3800 m and damaged the well casing. The slip in the fault plane and associated damage zone enhanced the porosity and permeability, eventually draining the existing water in PX-2. We overlapped the distribution of M > 2 aftershocks projected onto the assumed fault plane (Fig. 5a). The two largest earthquakes were located on the edges or out of the rupture area, and some aftershocks were located between two major slip patches, although other earthquakes were located throughout the slip distribution. This can be attributed to the complex fault geometry, heterogeneity of stress field, and the uncertainty of the inverted rupture model. In the acoustic image log of PX-1, the logging sensor moved downward to 4097 m from the surface, and the iron well casing in PX-1 did not exhibit any damage. However, in PX-2, the logging sensor only penetrated to 3800 m and was unable to reach the well bottom. This indicated that the well casing was damaged or most likely torn and dislocated at a depth of 3783 m (at a measured depth along the well trajectory; hereafter referred to as MD), which is 425 m above the well casing bottom at 4208 m (MD). Acoustic imaging of PX-2 exhibited that clay and muddy fluid plugged the well casing and prevented the logging sensor from moving downward. The open-hole section corresponds to 4208–4348 m (MD) in PX-2, such that it is almost impossible for the clay material to have moved upward to approximately 400 m (from 4208 to 3820 m) from the open-hole section. Geologic logging data also suggest that fault gouge was present at a depth of 3800 m in PX-2^2^. In addition, a large amount of loss circulation occurred at this depth. This indicated that clay material originated from the fault gauge and/or lost circulation mud moved into the broken section of the well after the M_W_ 5.5 earthquake as the well drilling caused a high amount of drilling loss circulation at that depth (Fig. 2).

### Hydrochemical and microbial changes after key seismic events

**Figure 6:**
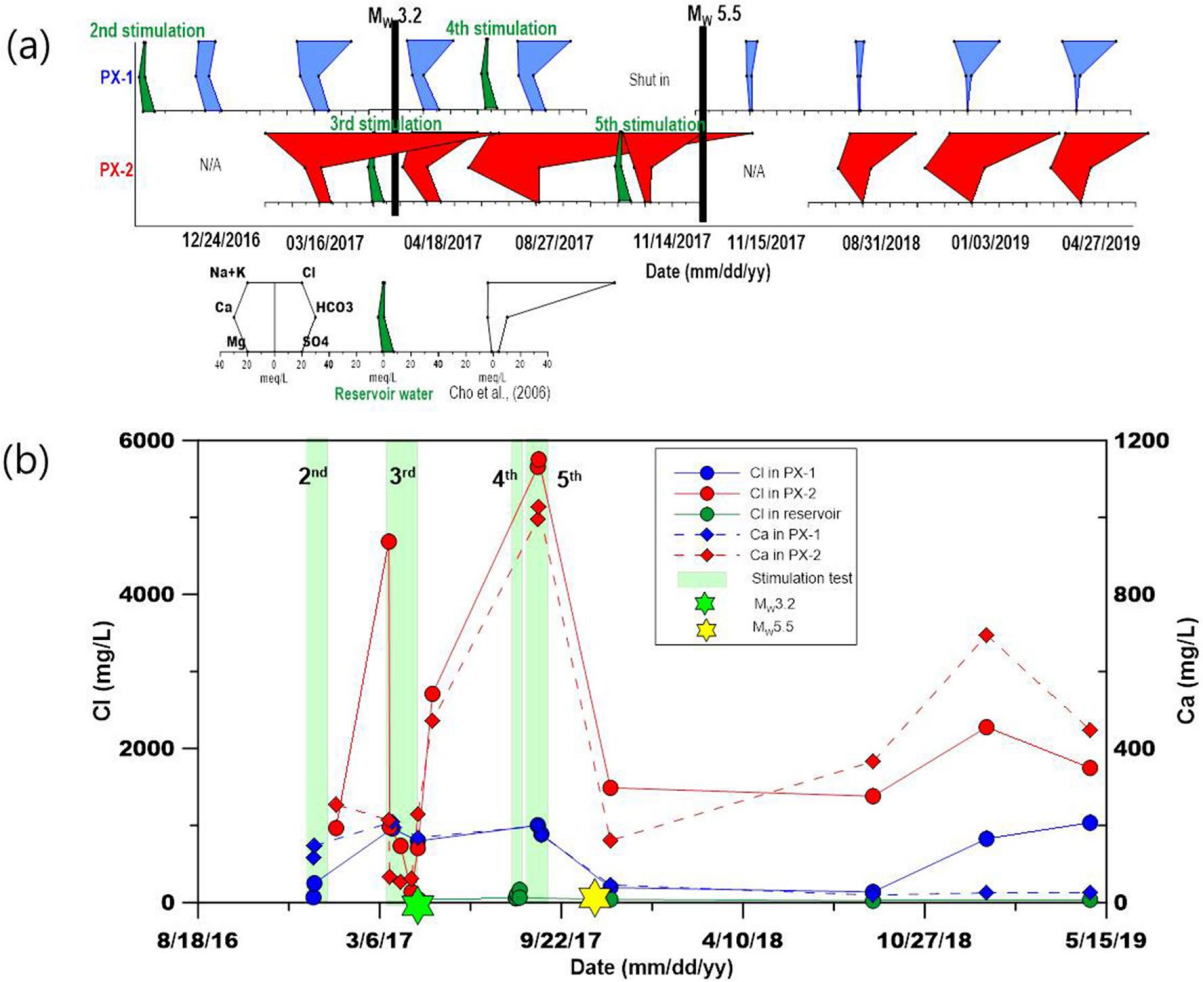
Stiff diagram (**a**); the surface reservoir water used for stimulation was indicated by green stiff diagrams; data collected on 27 August 2017 were modified from Burnside et al.^17^; and temporal variations of Ca and Cl concentrations in PX-1 and PX-2 after M_W_ 3.2 and M_W_ 5.5 earthquake (**b**).

After the M_W_ 5.5 earthquake, the hydrogeochemical properties of waters from PX-1 and PX-2 were distinctly different, as they were separated by 630 m. The water temperatures at the top of the PX-1 and PX-2 well columns were 25.4 and 44.5 °C, respectively after the M_W_ 5.5 earthquake (Fig. 3b). The δ^18^O and δD values in PX-1 and PX-2 increased when compared to those of water samples collected after the M_W_ 5.5 earthquake, with both samples deviating from the LMWL (Fig. 4a). In PX-2, the values of δ^18^O and δD showed larger variations than those of PX-1, possibly reflecting the effect of the earthquake. In PX-1, after the M_W_ 5.5 earthquake, the concentrations of Na, K, Cl, and SO_4_ decreased compared to those observed after the M_W_ 3.2 earthquake while the chemical species in PX-2 exhibited concentrations similar to those of the samples collected after the M_W_ 3.2 earthquake (Fig. 6a). The concentrations of Cl and Ca showed large variations after the M_W_ 5.5 earthquake. Time series data for the Cl and Ca concentrations before and after the M_W_ 3.2 and 5.5 earthquake are presented in Fig. 6b. The concentrations of Cl showed extremely large variations in PX-2, indicating abrupt changes in the hydrogeochemical conditions caused by different environments after the M_W_ 5.5 earthquake.

The concentrations of radioactive dissolved inorganic carbon (^14^C_DIC_) exhibited distinct differences between PX-1 and PX-2, corresponding to 46.69 and 16.38 pmC, respectively, after the M_W_ 5.5 earthquake, although the ^14^C_DIC_ concentrations were similar to the concentrations corresponding to 27.23 and 26.16 pmC in PX-1 and PX-2, respectively, after the M_W_ 3.2 earthquake (Fig. 4b). The concentrations of ^14^C_DIC_ in PX-1 after the M_W_ 5.5 earthquake increased when compared to those of samples collected after the M_W_ 3.2 earthquake while the groundwater in PX-2 exhibited decreased concentrations of ^14^C_DIC_ (Fig. 4b). The increased concentrations of ^14^C_DIC_ in PX-1 indicated that the water was affected by younger reservoir water. The values of δ^13^C in PX-1 and PX-2 also exhibited a result consistent with the concentrations of ^14^C_DIC_. Similar values for δ^13^C in PX-1 and PX-2 after the M_W_ 3.2 earthquake changed to different values following the M_W_ 5.5 earthquake.

The water levels rose approximately to 13.7 and 35.9 m in PX-1 and PX-2, respectively, during the six months of monitoring from August 30, 2018, to February 28, 2019 (Fig. 3b). The concentrations of Na, K, and Cl increased in PX-1 over time after the M_W_ 5.5 earthquake. The concentrations of Ca and Cl significantly increased in PX-2 when compared to those in PX-1 (Fig. 6a). Given that geothermal water in South Korea exhibits high concentrations of Na and HCO_3_^18,36^, increased concentrations of Na, Cl, and HCO_3_ in PX-2 suggested that the formation water entered PX-2 through the ruptured casing located along the ruptured fault plane. The ruptured well casing of PX-2 caused fundamental differences in the water chemistry between PX-1 and PX-2 following the main shock on November 15, 2017.

**Figure 7:**
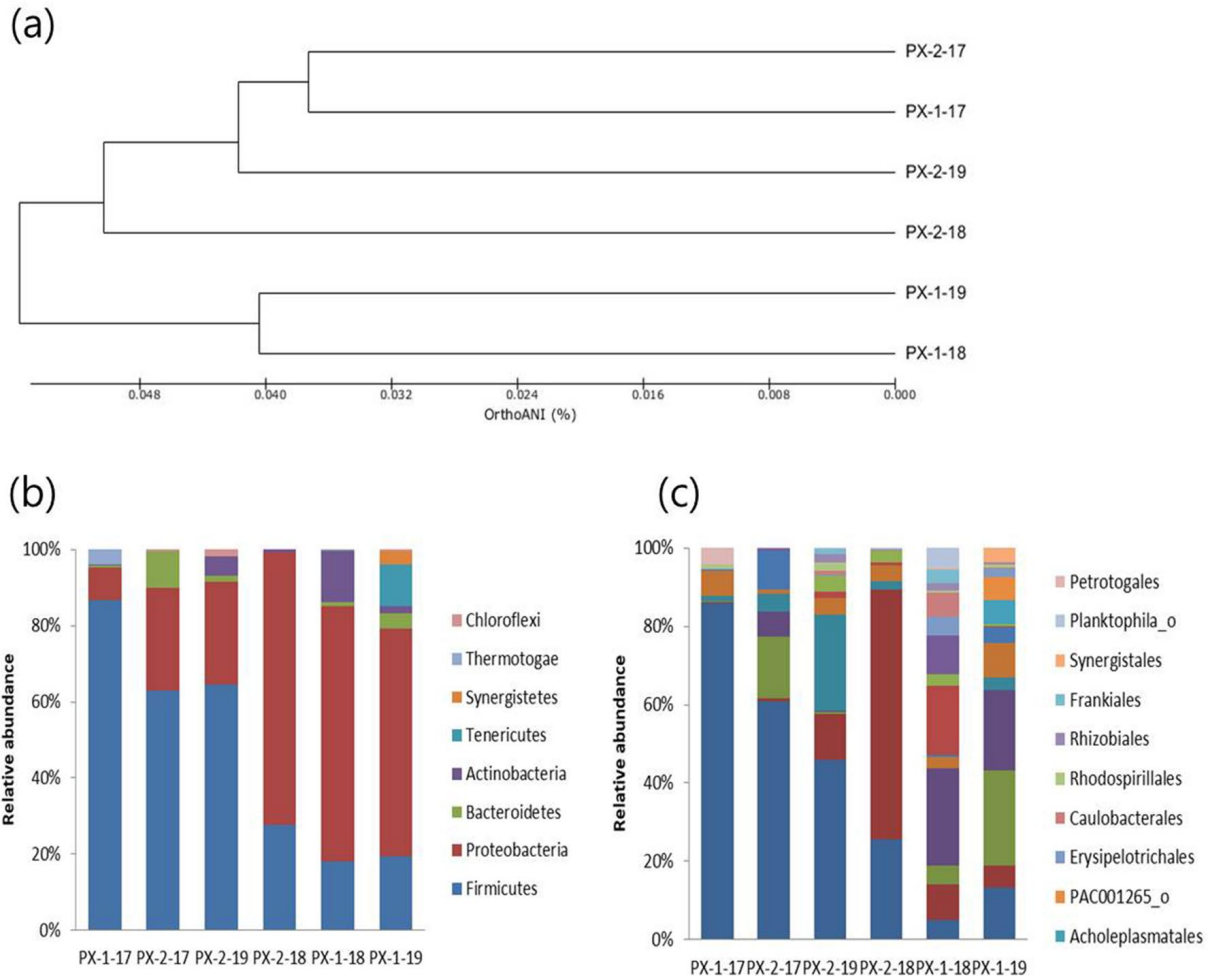
(**a**) Hierarchical clustering of bacterial communities in PX-1 and PX-2 sampled after the M_W_ 3.2 (PX-1–17 and PX-2–17) earthquake, M_W_ 5.5 earthquake in August 2018 (PX-1–18 and PX-2–18), and in April 2019 (PX-1–19 and PX-2–19). Relative sequence abundances of bacteria taxa in PX-1 and PX-2 at the (**b**) phylum and (**c**) order levels.

The structures of bacterial communities also showed results that were consistent with the hydrochemical data. Microbial community structures in PX-1 and PX-2 sampled after the M_W_ 3.2 (PX-1–17 and PX-2–17) and 5.5 earthquakes (PX-1–18 and PX-2–18) were compared at the phylum and order levels using 16S rRNA gene-based pyrosequencing (Fig. 7). Hierarchical clustering indicates that the bacterial communities in PX-1–18 and PX-2–18 after the M_W_ 5.5 earthquake exhibited different structures while PX-1–17 and PX-2–17 after the M_W_ 3.2 earthquake exhibited similar microbial community structures (Fig. 7a). The bacterial communities in PX-1–19 and PX-2–19, sampled in April 2019, also exhibited different structures. The bacterial communities in PX-1 and PX-2 after the M_W_ 5.5 earthquake belonged to different groups, suggesting that they originated from different aquifer conditions.

### Wavelet analyses of the water level data

After the rapid drop in the water levels was confirmed in the two EGS wells, changes in the water levels were measured at intervals of 10 min using an automatic water level recorder. A peculiar phenomenon was observed in the water level variation in which a distinct semi-diurnal cyclic fluctuation appeared in PX-1 while no such variation was observed in PX-2. Earth tides can change the pore pressure and thus the groundwater level. Synthetic Earth tides at the Pohang area showed an exact correlation with the water level fluctuation in PX-1. Figure 3c shows a time series of Earth tide vertical components and the responses of each well to the Earth tide. The presented water levels were corrected for atmospheric pressure changes. The Earth tide has 20–30 cm vertical variations and the water level in well PX-1 reversely responded to the Earth tide with 2–3 cm variations. The reverse response is due to the positive “up” component of the Earth tide, which causes an expansion of the aquifer and reduces the fluid pressure, thus causing the water level drop. As shown in Fig. 3c, no noticeable response in the water levels of well PX-2 indicates that well PX-2 lost its hydraulic connection to the deep groundwater. The well served as a long one-ended pipe storing wellbore water, such that the Earth tide could not influence the water level.

**Figure 8:**
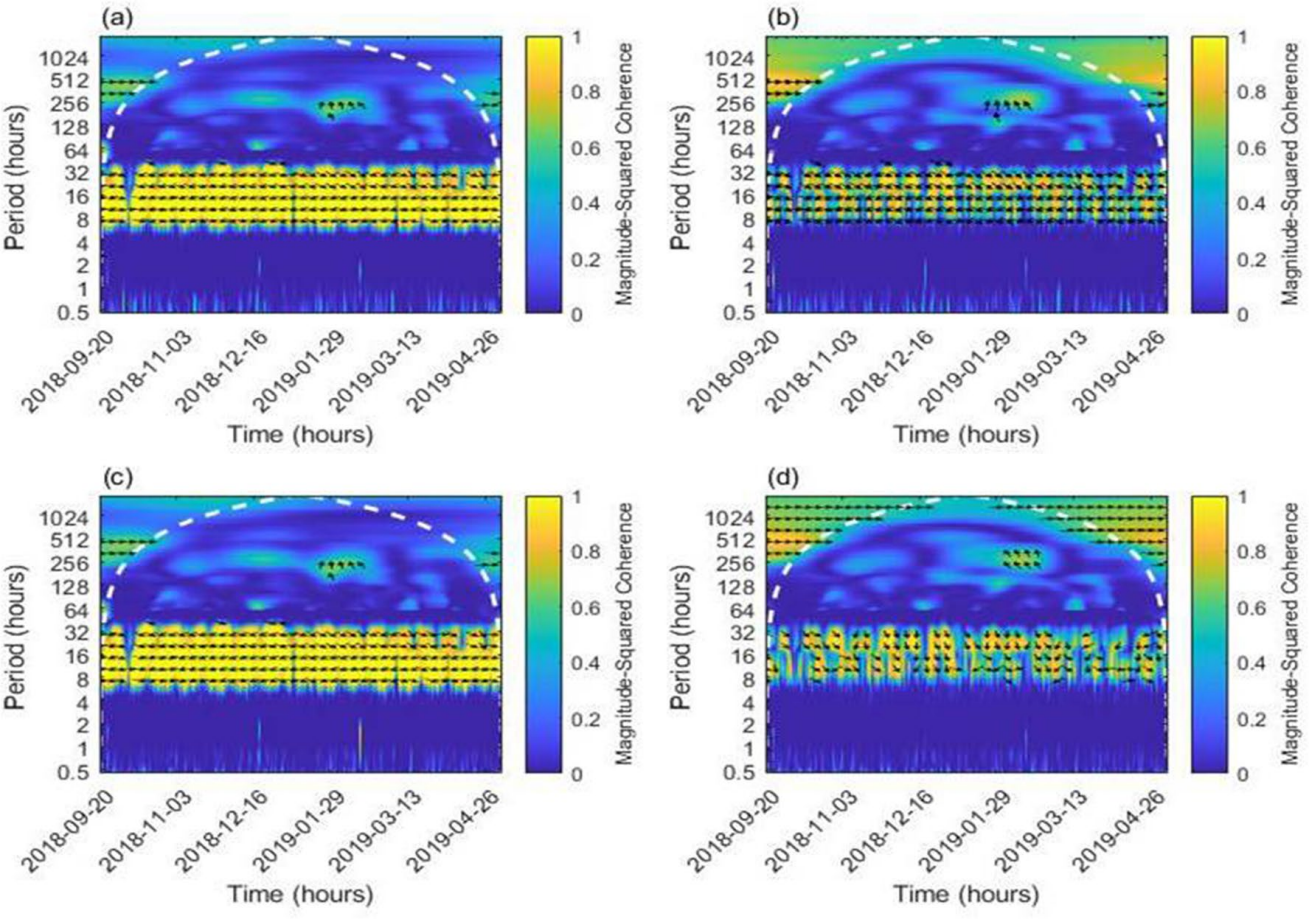
Wavelet coherence between the Earth tide and detrended PX-1 water level (**a**); detrended PX-2 water level (**b**); detrended and denoised PX-1 water level (**c**); and detrended and denoised PX-2 water level (**d**). Possibly distorted areas are divided with white dotted lines.

Figure 8 shows the wavelet analyses of the water level data and Earth tide data. The Earth tide showed a high coherence with the water level of well PX-1, but not in PX-2 in the range of 8 to 32 h periods. Wavelet analyses were also applied to two national groundwater monitoring wells near the EGS site, i.e., the Sin-gwang and Yeon-il sites. The processes were the same as for the Pohang site and the result is shown in Fig. S3. A good coherence was also observed between the Earth tide and the water level of well PX-1. When compared with Fig. 8, PX-1 shows the highest coherence and PX-2 shows the lowest coherence with the earth tide by comparing the area of the 8 to 32 h periods.

## Discussion

Following the M_W_ 5.5 earthquake, PX-1 exhibited similar groundwater levels, i.e., 95–100 m below the ground surface, with other deep wells near the EGS site^37^ while PX-2 exhibited an extremely deep water level of 740 m. In PX-1, 1695 m^3^ of water remained after 5663 m^3^ of water was injected for two hydraulic stimulations with 3968 m^3^ of water that flowed back. In contrast, 4146 m^3^ of water remained in the aquifer around PX-2 after 7135 m^3^ of water was injected and 2989 m^3^ was discharged (Fig. S1). Following the M_W_ 5.5 earthquake, 453 m^3^ of water flowed back from well PX-1. Thus, the water in PX-1 should be a mixture of formation water and injected water. The net injection volume in PX-2 is significantly larger than that in PX-1, but the flowback water properties in PX-1 were more significantly affected by injected surface reservoir water when compared to the flowback water in PX-2. This is because water in PX-2 was affected by the formation water that entered PX-2 through the ruptured well casing at a depth of 3800 m, i.e., where the fault slip crossed the PX-2 well casing. Formation water near a depth of 3800 m entered PX-2 with mud, causing PX-2 to exhibit similar hydrochemical characteristics to those of the formation water. Given the intrusion of mud with the formation water, the interior of the casing near the 3800 m depth was clogged with mud, thereby exhibiting extremely low permeability. Thus, the water level in well PX-2 slowly recovered at a rate of approximately 5.93 m/month (Fig. 3b).

The injected water and formation water exhibited distinctive hydrochemical properties. The mixing ratios between the injected water and formation water were calculated using two elements, strontium isotopes and chloride, and a binary mixing ratio in Eqs. 2–4 in the supporting materials. Table 1 lists the results of the calculated mixing ratios for each sampling time. Only chloride data existed prior to the M_W_ 5.5 earthquake as a conservative tracer. With respect to the chloride concentrations, the mixing ratio or ratio of surface reservoir water to water bled out from PX-1 corresponded to 13.01% before the main events, 1.99% nine months after the main events, 14.03% 14 months after the main events, and then recovered to 17.49% after 17 months. The results using the strontium isotopes exhibited lower values when compared with the chloride ions because the Sr isotopes are more stable. However, this also indicated that the values increased from August 2018 to April 2019. Furthermore, the mixing ratios of PX-1 based on the two elements were extremely low in August 2018, which is similar to the values for PX-3 (reservoirs) based on the results from the Stiff diagram (Fig. 6a).

**Table 1:** Calculated mixing ratios of the formation water in the well (%).

Well ID	18 April 2017		31 August 2018		28 January 2019		27 April 2019	
	Sr	Cl	Sr	Cl	Sr	Cl	Sr	Cl
PX-1	–	13.01	1.0	1.99	4.8	14.03	3.0	17.49
PX-2	–	22.3	48.8	23.7	100	39.3	61.8	29.9

The changes in the mean stress (i.e., (σ_11_ + σ_22_ + σ_33_)/3) from the finite rupture model of the Pohang mainshock were investigated to verify whether the decreased ground water levels corresponded to the co-seismic deformation (Fig. 5a). We estimated the distribution of the stress drop in the fault model (Fig. 5b) and the changes in the mean stress with depth for wells PX-1 and PX-2 (Fig. 5c,d). Considering that the relative location between the wells and the slip model may have uncertainties, we further examined the changes in the mean stress at peripheral areas of the wells, horizontally shifting their trajectories by – 200 to 200 m with intervals of 100 m in both the EW and NS directions. The mean stress for well PX-2 significantly varied near 3.5 km, which corresponds to the crossing points of the well and assumed fault geometry. The mean stresses at the middle of the open hole interval in wells PX-1 and PX-2 were changed by 0.5 and – 0.3 MPa, respectively.

When the normal stress changes were fully converted to the pore pressure changes, the water level difference between the two wells was approximately less than 100 m while PX-2 had a subsequent drop in the water table. However, the modeling results, considering only the stress changes around the mainshock rupture, were far from the observed significant water level drop. Hence, another possible mechanism needs to be determined to explain the sudden water level drop. We proposed a mechanism based on the PX-2 casing rupture and mud clogging observed through borehole acoustic image logging 2. Before the Pohang earthquake, the wellbores of PX-1 and PX-2 were filled with water to the top of the casing as the wells spilled water to the surface. The Pohang earthquake mainshock ruptured the casing of PX-2 at a depth of approximately 3800 m, where the fault crossed the well. Wellbore water in PX-2 was discharged through the ruptured casing toward the ruptured fault where the pore pressure suddenly decreased and the porosity increased because of the rupture. Flow into the ruptured zone gradually increased the pore pressure along the ruptured fault and finally caused the pore pressure to be equilibrium with that of the rock formation near the ruptured fault causing the fluid pressure gradient to reserve toward the wellbore. The reserved pore pressure gradient forced the formation water flow back into the wellbore through the ruptured casing. However, flowback into the wellbore was hindered by the very low-permeable mud pile at the ruptured well casing because the well casing at an approximate depth of 3800 m was ruptured and dislocated with the mainshock fault slip; fault clays were pushed into the well and caused blockage when the pore pressure gradient was reserved into the wellbore. As a result, a cylindrical low permeability section formed near the ruptured well casing. This is the reason the water level has been recovering very slowly. Based on a simple application of Darcy’s law, the approximated hydraulic conductivity of the low-permeable cylindrical section is of the order of 10^–6^–10^–5^ cm/s, which is generally the hydraulic conductivity of silt and silty clay^38^.

**Figure 9:**
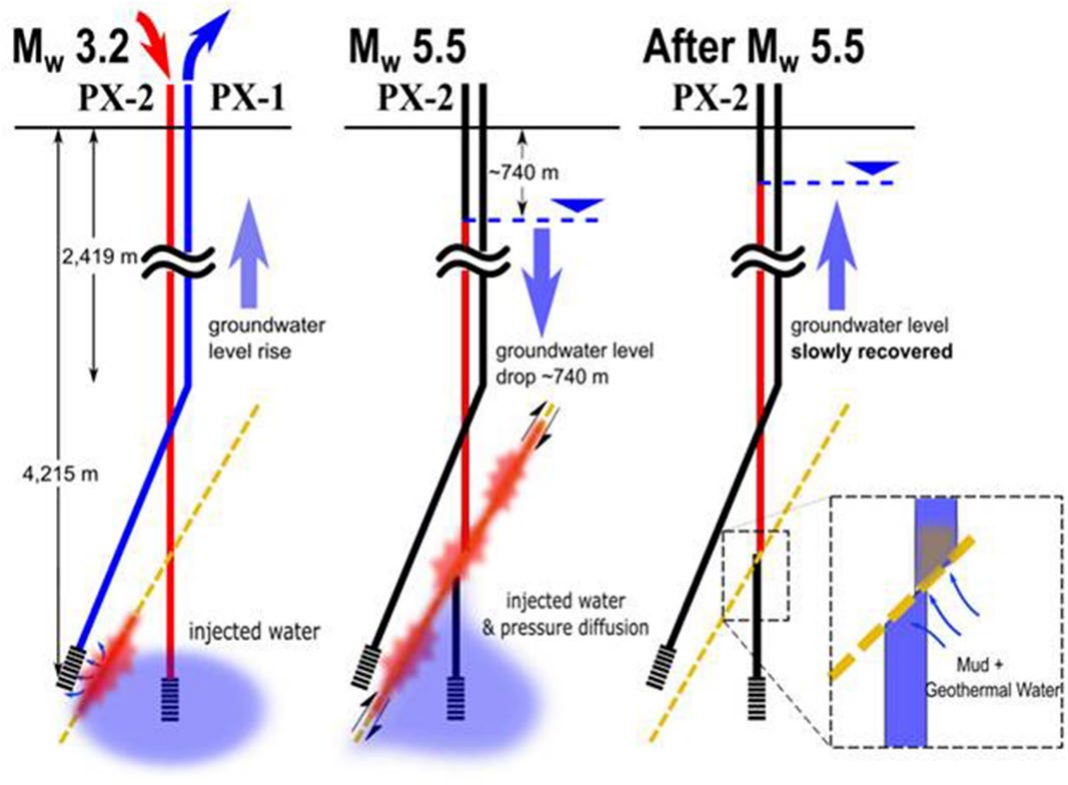
Schematic illustration of groundwater level changes after the M_W_ 3.2 and M_W_ 5.5 earthquakes.

The data analysis presented in this study allows the inference of two unique events of fluid inflow in the fault zone before and after the main shock (Fig. 9). The first event caused microearthquakes, whereas the second event (co- or post-seismic) was accompanied by direct water inflow into the fault. A unique outflow event occurred during the M_W_ 3.2 earthquake on April 15, 2017, wherein PX-1 discharged formation water containing dissolved gases. The fact that formation water was discharged through PX-1 indicated that the M_W_ 3.2 earthquake (induced by PX-2 stimulation) created a hydraulic connection between a small-sized trapped geothermal reservoir and the open hole section of PX-1. After the M_W_3.2 earthquake, observations of the hydrochemical differences between the two wells suggest the occurrence of some subsurface conditions that should be recognized as unusual or due to the existence of a fault; therefore, operators should be alerted for potential risks of future earthquakes.

This study revealed the hydraulic, chemical, and microbial variations in deep formation water regions as observed through deep geothermal wells. The circulation mud loss event confirmed that a few hundred cubic meters of fluid injection under an excessive pore pressure of ~ 20 MPa by itself can induce a swarm of earthquakes if the fluid is directly injected into the fault zone occuring a near critically stressed state. The water quality observations from the M_W_ 3.2 earthquake indicated that fault movement disturbed the surrounding formation water reservoir and resulted in the mixing of deep groundwater and a change in the temperature–pressure conditions. The extreme water level drop observed in well PX-2 after the M_W_ 5.5 main shock and associated changes in the chemical, isotopic, and microbial signatures of water led to valuable observations and implications on co- and post-seismic pore water pressures due to faulting at a depth of 4 km. This study also highlights the value of real time monitoring and analyzing the water chemistry, in addition to seismic monitoring during EGS operation to prevent earthquakes. This study revealed the first extreme hydrological responses through induced earthquakes and provides new insights into multiple mechanisms caused by hydrological, geochemical, and microbial responses to induced earthquakes in the deep aquifer system. Quantitative and thorough analyses of the more than 740 m-water level drop in PX-2 after the Pohang Earthquake still requires further research.

## Material and methods

### Site description

The Pohang EGS site is located in Pohang, South Korea (Fig. 1). Two EGS wells (PX-1 and PX-2) were drilled at the Pohang EGS site in the Heunghae Basin, which is covered by a thin Quaternary alluvium deposit underlain by 200–400 m thick Tertiary mudstone, sequential 1000 m thick Cretaceous sediments, and 900 m thick Cretaceous volcanics on the Permian granodiorite^39^. PX-1 and PX-2 are 6 m apart on the ground and approximately 600 m into the open-hole sections at the bottom level of 4,300 m. The measured depths correspond to 4,362 and 4,348 m, respectively, in PX-1 and PX-2 (Fig. 1). PX-1 was installed in 2013 to a depth of 4127 m; the drill pipes were then broken and stuck in the borehole. As a result, PX-1 was deviated from vertical drilling with an inclination of 21.5° from 2419 m to the final depth of 4361.8 m. Figure 1 shows the schematic diagrams of the two wells.

Water from a surface reservoir at a distance of 500 m from the EGS site was used for injection into PX-1 and PX-2 during five hydraulic stimulations. Hydraulic stimulations were conducted twice in PX-1, from December 15 to 28, 2016 and August 7 to 14, 2017. Hydraulic stimulations were conducted three times in PX-2, from January 29 to February 20, 2016; March 16 to April 14, 2017; and August 30 to September 18, 2017. The volume of water injected into and flowing back from PX-1 and PX-2 are listed in Table S2 and shown in Fig. S1. The accumulated injection volumes in PX-1 and PX-2 corresponded to 5663 and 7155 m^3^, respectively, and the accumulated back water flow volume from PX-1 and PX-2 corresponded to 3968 and 2989 m^3^, respectively (Fig. S1). The maximum wellhead pressure and injection rate were 27.7 MPa and 19.08 L/s in PX-1. A slightly higher maximum wellhead pressure (89.2 MPa) and injection rate (46.8 L/s), when compared to those for PX-1, were applied to PX-2.

### Acoustic image logging and groundwater monitoring

Acoustic images of PX-1 and PX-2 were obtained to detect any possible damage to the casing and open-hole sections of PX-1 and PX-2 after the M_W_ 5.5 main shock. Acoustic image logging was conducted by HADES with a QL43 ABI 2G to survey the 7″ casing and into the 8–1/2″ open-hole sections. After the abrupt water level drop in PX-2 during the earthquake was confirmed and the existence of a significant water level difference was observed during acoustic image logging, water level and temperature monitoring commenced in August 2018 (Fig. 3). The water level and temperature in the EGS wells were measured every 10 min by automatic recording loggers (TD-level logger, Van Essen). Level loggers were installed at approximately 40 m below the water level and at depths of 144 and 780 m from the drilling floor in PX-1 and PX-2, respectively.

### Water sampling procedure

Water samples were collected in April 2017 after the M_W_ 3.2 earthquake and in August 2018, as well as in January and April 2019 after the M_W_ 5.5 earthquake. In PX-2, the groundwater that flowed back was sampled immediately before the M_W_ 5.5 earthquake. In PX-1, groundwater sampling immediately after the M_W_ 5.5 earthquake was also feasible because groundwater flowed back after the earthquake and was stored in a water tank. Groundwater in PX-2 did not flow back to the surface because the fault crossed PX-2 and the water level decreased by more than 740 m. The fault rupture during the M_W_ 3.2 earthquake did not cross PX-1 and PX-2 and groundwater flowed back in both wells; thus, water samples were collected from the flow cell using the tube installed inside the wells. Following the M_W_ 5.5 earthquake, it was difficult to access the EGS site due to problems related to the safety check. When all the safety checks around the EGS site were completed, groundwater samples were collected in August 2018 using bailers because the water levels had decreased to 113 and 740 m below the drilling floor; thus, it became highly difficult to collect the groundwater using a submersible pump.

### Chemical analysis

Cation and anions were analyzed at the Korea Basic Science Institute (KBSI) using inductively coupled plasma atomic emission spectrometry (ICP-AES) and ion chromatography. The isotope values of δ^18^O and δD were measured at KBSI by a VG PRISM II stable isotope ratio mass spectrometer. In addition, ^87^Sr/^86^Sr ratios were measured by a Neptune Multicollector-Inductively Coupled Plasma Mass Spectrometer (MC-ICP-MS; Thermo Finnigan, Germany) at KBSI and were normalized to ^87^Sr/^86^Sr = 0.1194^40^, and the mean ^87^Sr/^86^Sr ratio of NBS987 (U.S. National Bureau of Standards) corresponded to 0.710247 ± 0.000017 (2σ, n = 18). The values of the δ^13^C and ^14^C concentrations of DIC were analyzed at the University of Waterloo via a stable isotope mass spectrometer (Thermo Scientific DeltaVplu) and an accelerator mass spectrometer. δ^13^C was measured in per mil (‰) relative to the Pee Dee Belemnite ratio. Percent modern carbon (pmC) was used as the unit for the ^14^C measurements.

### Microbial analysis

The microbial community structures in the water from PX-1 and PX-2 sampled after the M_W_ 3.2 earthquake (PX-1–17 and PX-2–17) and the M_W_ 5.5 earthquakes in August 2018 (PX-1–18 and PX-2–18) and April 2019 (PX-1–19 and PX-2–19) were analyzed via 16S rRNA gene-based pyrosequencing. Water samples were filtered via a 0.2 μm filter in the field and stored in a refrigerator (– 70 °C). Subsequently, DNA was extracted via a Fast DNA spin Kit (Qbiogen, USA). The extracted DNA was amplified via forward and inverse primers to distinguish each sample^41,42^. Pyrosequencing was conducted via a 454 GS junior sequencing system (Roche, NJ, USA) by Chun Laboratory (Seoul, Korea). Operational taxonomic units (OTUs) were used to determine bacterial community structures and calculate the abundance-based coverage estimator (ACE), Chao 1 richness estimator, Shannon and Simpson diversity indices, and rarefaction curves. Each sequence was analyzed and compared with sequences in the EzTaxon-extended database (Chun Lab, eztaxon-e.org) via BLASTIN searches and pairwise similarity comparisons. The full sequences were analyzed and compared with other known sequences that were available in the NCBI (National Center for Biotechnology Information) database.

### Wavelet analysis

One of the visualizing methods to analyze the tide effect on the wells is to generate a wavelet spectrogram of the tide and water level^43^. Using MATLAB, we obtained the period-time wavelet power spectrogram based on the Morse wavelet, showing the coherence of the tide and water level data. When compared to the Fourier transforms, the importance of the wavelet transforms is that they partially overcome the uncertainty principle via multi-resolution decomposition^44,45^. By taking advantage of this, the wavelet power spectrogram showed the period of the data, which exhibited high coherence and provided an opportunity to confirm whether the high coherence part was the main composition of the resource data. In this study, tide data, which were mainly composed of diurnal (period of 24 h) and semidiurnal (period of 12 h) tides, were the resource data for the spectrogram. The major focused part of the spectrogram was the scope, including the 12 and 24 h periods. Instead of sea tide data, the used Earth tide data are more detailed to the exact location (Pohang site). In addition, as PX-1 and PX-2 are recovering wells, the water level data for PX-1 and PX-2 were linearly detrended. To compare the results with the clearance of noises, noise filtering process with the discrete wavelet transform, based on the “Daubechies 7” function, Bayes method, level 12, and median rule, was also performed before manually generating the spectrogram on the MATLAB “Wavelet Signal Denoiser” toolbox to clearly observe the effect of the tide on the wells.

### Finite rupture model

Kinematic source parameters of the finite fault models were estimated based on the surface deformation measured from the Differential Interferometry Synthetic Aperture Radar and local seismograms, whose epicentral distances are less than 60 km. The assumed geometry of the fault model was determined from the principal component analysis for the aftershock distribution (strike: 214°, dip: 63°). Static deformation generated from the cumulative slip distribution was calculated from the analytic solution reported by Okada^46^ in a homogeneous half-space model.

## Supplementary Information

Supplementary Information.
