# Supplementary material for: Earthquakes and very deep groundwater perturbation mutually induced
Source: Sci Rep. 2021 Jul 1;11:13632. doi: 10.1038/s41598-021-92937-y (PMC8249596; doi:10.1038/s41598-021-92937-y)
Supplement: Supplementary file 1 — Supplementary Information. [file 41598_2021_92937_MOESM1_ESM.pdf]

**Supplementary Information for**

# Earthquakes and very deep groundwater perturbation mutually induced

Dugin Kaown[1], Kang-Kun Lee[1]*, Jaeyeon Kim[1], Jeong-Ung Woo[2], Sanghoon Lee[1], In-Woo Park[1], Daeha Lee[1], Jin-Yong Lee[3], Heejung Kim[3], Shemin Ge[4], In-Wook Yeo[5]

[1]School of Earth and Environmental Sciences, Seoul National University, Seoul 08826, Korea

[2]Department of Geophysics, Stanford University, Stanford, CA 94305, USA

[3]Department of Geology, Kangwon National University, Chuncheon 24341, Korea

[4]Geological Sciences, University of Colorado, Boulder 80309, USA

[5]Department of Geological and Environmental Sciences, Chonnam National University, Gwangju 61186, Korea

*Kang-Kun Lee

**Email: kklee@snu.ac.kr**

**This PDF file includes:**

Figures S1 to S3

Tables S1 to S2

Table S1. The seismicity with the mud loss data in the study site.

| **Mud loss data** | | **Seismicity data** | | | | | |
|---|---|---|---|---|---|---|---|
| Date (KST) | Total mud loss ($m^3$) | Origin time (KST) | | Magnitude | Event ID | Status | |
| 2015-09-22 | 15.00 | 2015-11-01 | 13:33:26.450 | -0.054 | 201511010433a | NE | NLOC |
| 2015-09-24 | 17.59 | 2015-11-01 | 20:14:01.489 | -0.285 | 201511011114a | NE | NLOC |
| 2015-10-27 | 3.52 | 2015-11-03 | 00:33:31.279 | -0.440 | 201511021533a | NE | NLOC |
| 2015-10-30 | 25.10 | 2015-11-03 | 18:12:19.699 | -0.261 | 201511030912a | NE | PHA2 |
| 2015-10-31 | 134.30 | 2015-11-05 | 03:48:02.068 | -0.684 | 201511041848a | NE | PHA2 |
| 2015-11-01 | 29.00 | 2015-11-05 | 13:44:15.300 | -0.048 | 201511050444a | NE | PHA2 |
| 2015-11-02 | 20.40 | 2015-11-05 | 20:31:20.099 | -0.423 | 201511051131a | NE | PHA2 |
| 2015-11-03 | 59.10 | 2015-11-05 | 22:18:51.379 | -1.015 | 201511051318a | NE | PHA2 |
| 2015-11-04 | 27.90 | 2015-11-09 | 09:36:59.980 | -0.101 | 201511090036a | NE | PHA2 |
| 2015-11-05 | 33.40 | 2015-11-15 | 07:49:50.600 | -0.101 | 201511142249a | NE | LOC |
| 2015-11-06 | 17.40 | 2015-11-19 | 06:32:33.300 | 0.350 | 201511182332a | NE | LOC |
| 2015-11-07 | 5.60 | 2015-11-22 | 16:59:43.689 | 0.603 | 201511220759a | NE | PHA2 |
| 2015-11-08 | 7.60 | 2015-11-26 | 11:12:51.460 | 0.177 | 201511260212a | NE | PHA2 |
| 2015-11-09 | 17.80 | 2015-11-30 | 12:52:17.690 | -0.267 | 201511300352a | NE | NLOC |
| 2015-11-10 | 20.20 | 2015-11-30 | 12:52:20.660 | 0.803 | 201511300352b | NE | LOC |
| 2015-11-13 | 46.60 | 2015-11-30 | 12:52:30.730 | -0.472 | 201511300352c | NE | NLOC |
| 2015-11-14 | 50.00 | 2015-12-01 | 19:27:09.289 | -0.148 | 201512011027a | NE | NLOC |
| 2015-11-18 | 34 | 2015-12-01 | 20:21:20.209 | -0.109 | 201512011121a | NE | NLOC |
| 2015-11-30 | 12 | | | | | | |
| 2015-12-04 | 10.8 | | | | | | |
| 2015-12-06 | 51.62 | | | | | | |

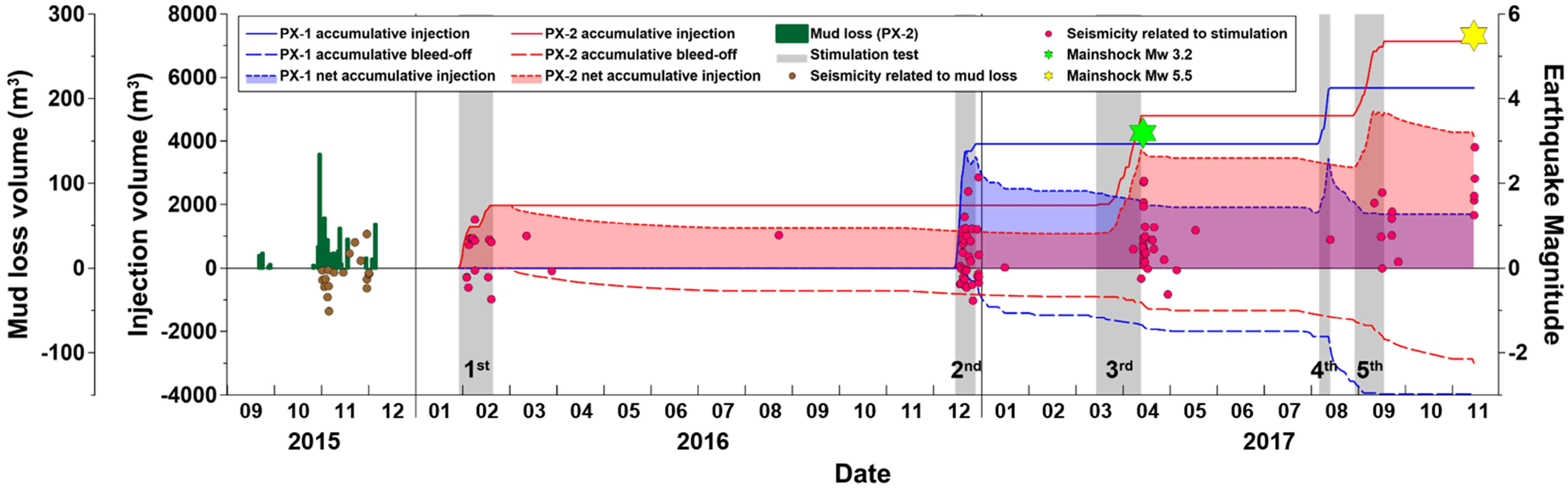


Fig S1. Accumulated injection volume and bleed-off during five hydraulic stimulations.

(a)

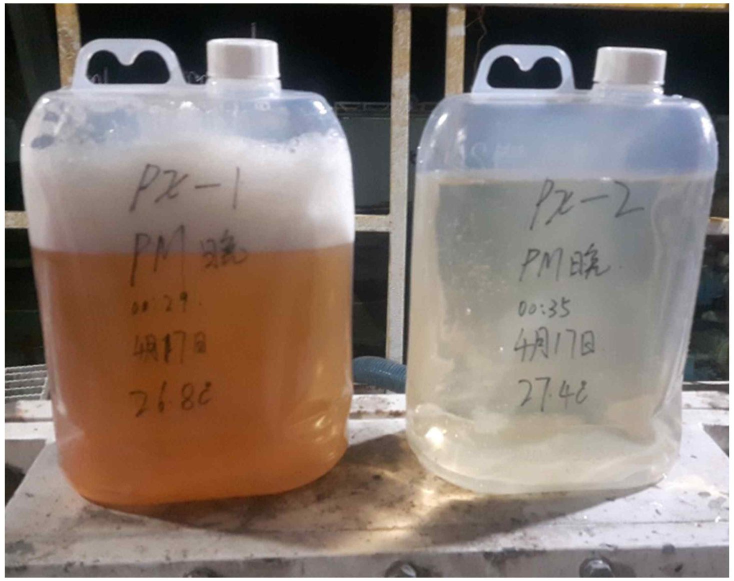


(b)

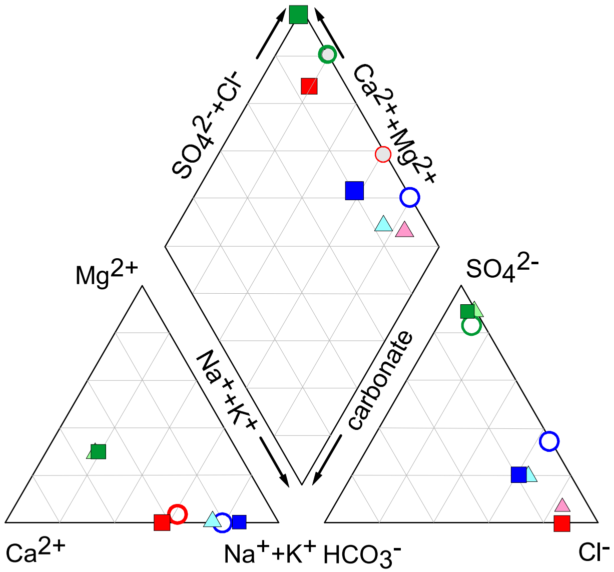


Fig. S2. Comparison of the water chemistry of PX-1, PX-2, and reservoir water used as injection water after the $M_W$ 3.2 and 5.5 earthquakes in (a) color (PX-1 and PX-2 sampled in April 17 at 26.8 and 27.4 °C, respectively) and (b) water type.

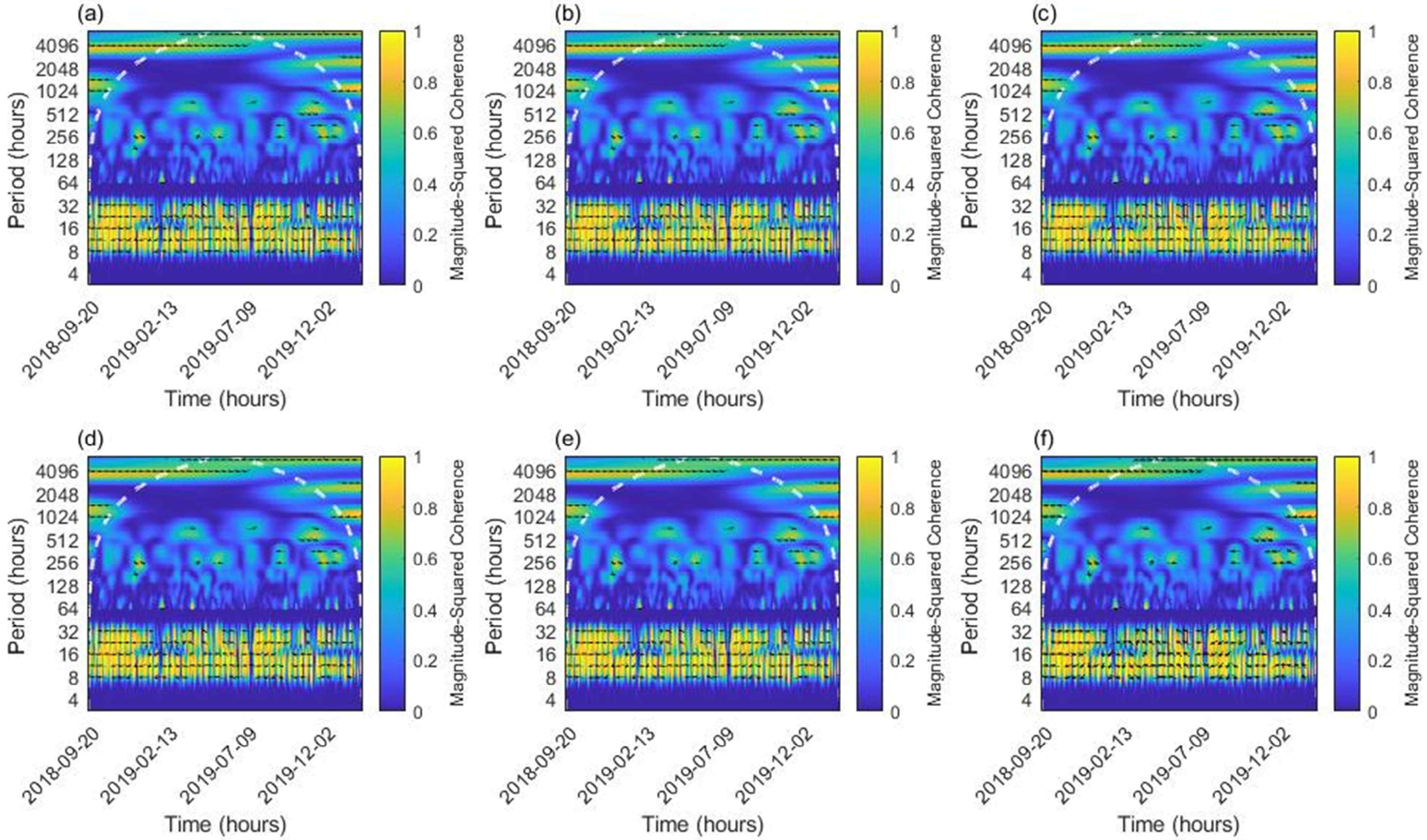


Fig. S3. Wavelet coherence between the earth tide and detrended Sin-gwang water level (a); detrended Yeon-il rock (water) level (b); detrended Yeon-il (alluvium) water level (c); detrended and denoised Sin-gwang water level (d); detrended and denoised Yeon-il (rock) water level (e); and detrended and denoised Yeon-il (alluvium) water level (f).

Table S2. Hydraulic stimulations event at PX-1 and PX-2

| Hydraulic Stimulation events | Injection well | Stimulation time | Injection Volume ($m^3$) |
|---|---|---|---|
| 1st | PX-2 | Jan. 29 to Feb. 20, 2016 | 1,969 |
| 2nd | PX-1 | Dec. 15 to Dec. 28, 2016 | 3,907 |
| 3rd | PX-2 | Mar. 16 to Apr. 14, 2017 | 2,832 |
| 4th | PX-1 | Aug. 7 to Aug. 14, 2017 | 1,756 |
| 5th | PX-2 | Aug. 30 to Sep. 18, 2017 | 2,334 |

## The hydraulic conductivity

Based on the water level recovery data measured since August 2018, the water level recovery rate of well PX-2 indicates a leaking condition in the pipe section plugged by the muddy fault materials. The hydraulic conductivity ($K$) can be estimated as follows:

$$K = \frac{Q}{\pi r_c^2}(\frac{\delta h}{\delta l}) = (\frac{\delta h}{\delta t})(\frac{\delta h}{\delta l}), \quad [1]$$

where $Q$ is the flux through the pipe, $r_c$ is the radius of the casing, $h$ is the head inside the well, and $l$ is the thickness of the plugged section. The head change rate and hydraulic head difference between the well and ambient groundwater were obtained from measured data. The thickness of the plugged section was assumed at a few meters. The hydraulic conductivity was then estimated to be $10^{-5}$–$10^{-6}$ cm/s, which indicates that the hydraulic conductivity of the silt in the silty clay matches the interpretation of the slow water level recovery in the well after a sudden water level drop due to casing rupture at the time of the earthquake.

**Mixing ratio**

The end members correspond to the highest value of the PX-2 and PX-3 samples (injected surface reservoir water). With respect to strontium isotopes, binary mixing ratios were calculated as follows:

$$(^{87}Sr/^{86}Sr)_M \times [Sr]_M = f((^{87}Sr/^{86}Sr)_{H.PX\text{-}2} \times [Sr]_{H.PX\text{-}2}) + (1-f)((^{87}Sr/^{86}Sr)_{PX\text{-}3} \times [Sr]_{PX\text{-}3}) \quad [2]$$

where $(^{87}Sr/^{86}Sr)_M$, $(^{87}Sr/^{86}Sr)_{H.PX\text{-}2}$, and $(^{87}Sr/^{86}Sr)_{PX\text{-}3}$ denote the $^{87}Sr/^{86}Sr$ ratios of each sample; and $[Sr]_M$, $[Sr]_{H.PX\text{-}2}$ and $[Sr]_{PX\text{-}3}$ denote the strontium concentrations of each sample (mg/L). The symbol f denotes the fraction of the highest value of the PX-2 samples. In addition, with respect to chloride concentrations, a simple mass balance equation for PX-1 and PX-2 waters was used as follows:

$$Q_m = Q_{FW} + Q_{IW} \text{ and} \quad [3]$$

$$C_m Q_m = C_{FW} Q_{FW} + C_{IW} Q_{IW}, \quad [4]$$

where $Q_{FW}$ and $Q_{IW}$ denote the discharges of each sample (formation water and injection water) to each sample, and $Q_m$ denotes the discharge from formation water and injection water ($m^3/s$). Furthermore, $C_m$, $C_{FW}$, and $C_{IW}$ denote the chloride concentrations of each sample, formation water, and injection water (mg/L), respectively. The endmember for the formation water was 5,753 mg/L, which was the highest concentration measured in PX-2 [1].

References

1 Burnside, N. M. *et al.* Rapid water-rock interactions evidenced by hydrochemical evolution of flowback fluid during hydraulic stimulation of a deep geothermal borehole in granodiorite: Pohang, Korea. *Applied Geochemistry* **111**, 104445 (2019).
